# Tumor Burden Measured by 18F-FDG PET/CT in Predicting Efficacy and Adverse Effects of Chimeric Antigen Receptor T-Cell Therapy in Non-Hodgkin Lymphoma

**DOI:** 10.3389/fonc.2021.713577

**Published:** 2021-08-04

**Authors:** Ruimin Hong, Elaine Tan Su Yin, Linqin Wang, Xin Zhao, Linghui Zhou, Guangfa Wang, Mingming Zhang, Houli Zhao, Guoqing Wei, Yiyun Wang, Wenjun Wu, Yafei Zhang, Fang Ni, Yongxian Hu, He Huang, Kui Zhao

**Affiliations:** ^1^Bone Marrow Transplantation Center, The First Affiliated Hospital, Zhejiang University School of Medicine, Hangzhou, China; ^2^Institute of Hematology, Zhejiang University, Hangzhou, China; ^3^Zhejiang Province Engineering Laboratory for Stem Cell and Immunity Therapy, Hangzhou, China; ^4^Liangzhu Laboratory, Zhejiang University Medical Center, Hangzhou, China; ^5^Positron Emission Tomography (PET) Center, The First Affiliated Hospital, Zhejiang University School of Medicine, Hangzhou, China

**Keywords:** chimeric antigen receptor T-cell therapy, non-Hodgkin lymphoma, metabolic tumor burden, FDG PET/CT, prognosis, adverse effects

## Abstract

Chimeric antigen receptor (CAR) T-cell therapy has exhibited promising clinical outcomes in treating relapsed/refractory (R/R) B-cell hematologic malignancies. Current studies have shown a close correlation between baseline tumor burden and therapeutic response in CAR-T cell therapy. However, the roles of PET/CT metabolic parameters, such as metabolic tumor volume (MTV) and total lesion glycolysis (TLG), remain unclear in this setting. In this study, we retrospectively reviewed 41 R/R NHL patients. 18F-FDG PET/CT was used to measure the average standardized uptake value (SUV_avg_), MTV, and TLG of the lymphomatous lesions. These patients were divided into two groups according to the optimal cutoff values of respective PET/CT metabolic parameters. The multivariate analysis depicted that early post-therapy SUV_avg_ (HR: 1.418, 95% CI: 1.029, 1.955; *p* = 0.033) and MTV (HR: 1.001, 95% CI: 1.000, 1.002; *p* = 0.041) were independent risk factors associated with OS and PFS, respectively. Patients with baseline SUV_avg_ < 4.36 achieved a superior 1-year OS rate than the SUV_avg_ ≥ 4.36 group (100.0% *vs.* 44.9%, *p* = 0.019). For the patients with lower values in early post-therapy SUV_avg_ (<2.60) (51.1% *vs.* 0%, *p* < 0.001), MTV (<0.55 cm^3^) (53.6% *vs.* 0.0%, *p* = 0.001), and TLG (<1.54) (53.6% *vs.* 0.0%, *p* = 0.001), their 1-year PFS rates were higher than the compared groups. Moreover, patients with higher baseline tumor burdens were found to have significantly increased CRS incidence and cytokine levels. In conclusion, the PET/CT metabolic parameters are closely related to OS, PFS, and CRS in R/R NHL patients treated with CAR-T cells. This study may pave the way for building a comprehensive assessment system of tumor burden using 18F-FDG PET/CT, which can optimize therapeutic and supportive approaches in CAR-T cell therapy.

## Introduction

Lymphoma, which can be classified as Hodgkin’s lymphoma (HL) and Non‐Hodgkin’s lymphoma (NHL), is a hematological malignancy that arises from B cells, T cells, or natural killer (NK) cells during different stages of maturation. Apart from conventional chemotherapy, the chimeric antigen receptor (CAR) T-cell therapy has recently emerged as an alternative therapy for relapsed/refractory aggressive B-cell lymphomas. The recent clinical reports demonstrated that the CD19 CAR-T cell therapy had made remarkable progress in achieving a complete remission (CR) rate of 50% in R/R diffuse large B cell lymphoma (DLBCL) patients (1, 2). However, the novel treatment of CD19 CAR-T cell treatment is often complicated by its adverse events such as cytokine release syndrome (CRS) (3). Also, the infiltration of activated CAR T cells into the tumor cells may influence molecular imaging findings such as PET/CT scan.

On the other hand, due to its sensitivity and specificity in determining the disease sites and tumor burdens, the 18F-fluorodeoxyglucose positron emission tomography-computed tomography (^18^F-FDG PET/CT) is widely used for the diagnosis, staging, and evaluation of response in patients with NHL (4–6). The FDG PET/CT ensures precise estimation of the anatomic extent, sizes, and volumes of the lymphomas. In this imaging technique, there are several prognostic factors and indicators used, namely, standardized uptake value (SUV), metabolic tumor volume (MTV), and total lesion glycolysis (TLG). To explain briefly, the SUV refers to a ratio of tissue radioactivity concentration and the injected dose against body weight; the MTV is a measurement of the tumor volume with a high metabolism, while TLG is defined as the product of the mean SUV and the MTV (7).

It is worth mentioning that the association between PET/CT metabolic parameters and the prognosis after chemotherapy in NHL, particularly DLBCL, has been intensively studied. Among these indicators, MTV is considered a better prognostic factor than SUV in solid tumors (8). Meanwhile, the TLG is considered a more accurate measure of disease burden than SUV_max_ (9). Furthermore, the baseline MTV and TLG are found to be correlated with the disease prognosis (10). A study conducted by Zhou et al. has suggested that both MTV and TLG can predict progression-free survival (PFS) and overall survival (OS) in DLBCL patients treated with R-CHOP therapy. A high TLG level is associated with dismal outcomes in PFS and OS (11). As far as we are concerned, merely little is known about the role of PET/CT in predicting the OS and PFS of lymphoma patients after CAR-T cell therapy. There were only limited studies to identify the predictive value of PET/CT parameters in adverse effects of CAR-T cell treatment (12, 13). Therefore, in this retrospective study, we utilized PET/CT prognostic indicators to evaluate post-CAR T-cell therapy’s treatment responses and toxicity in R/R NHL patients.

## Materials and Methods

### Patients and Data Collection

We retrospectively reviewed the clinical data of 41 relapsed/refractory NHL patients who received CD19 CAR-T cell therapy from April 2017 to November 2020 at the Bone Marrow Transplantation Center (The First Affiliated Hospital, School of Medicine, Zhejiang University). The inclusion criteria of this study were as follows: (1) diagnosed with R/R NHL; (2) treated with CD19 CAR-T therapy (3); performed ^18^F-FDG PET/CT before and after CAR-T cell infusion and evaluated by the PET Center (The First Affiliated Hospital, School of Medicine, Zhejiang University) (4); the follow-up data were complete and available. These patients’ data were collected from the digital imaging and electronic medical records system of the hospital. Furthermore, the diagnosis and CRS grading were identified based on the National Cancer Institute Common Terminology Criteria for Adverse Events (CTCAE) Version 5.0. The serum cytokine level (including IL-2, IL-4, IL-6, IL-10, TNF-α, IFN-γ, and IL-17A) was tested once a day at the Department of Clinical Laboratory during CRS. The study was registered on the ClinicalTrials.gov (NCT03118180).

### Treatment Protocol

The CAR structures, the manufacturing process, and CD19 CAR-T cell therapy protocols were reported previously (14). In summary, the mononuclear cells from the peripheral blood were collected from patients or healthy donors by leukapheresis. The anti-CD19 CAR-T cell were constructed with a 4-1BB costimulatory domain *via* a lentiviral vector. The manufactured CAR-T cell products were checked and strictly adhered to the China Medicinal Biotech Association’s code of conduct to ensure patient safety. Before the CAR-T infusion, all patients were conditioned with lymphodepletion chemotherapy regimens of fludarabine and cyclophosphamide. The baseline PET/CT data were evaluated after the last bridging treatment, if any, and before starting the lymphodepletion therapy, the early post therapy PET/CT were tested 1–2 month after CAR-T cell infusion.

### Response Assessment

Treatment response was assessed 1 month after CAR-T cell infusion according to the revised criteria for Response Assessment of the Lugano Classification (15). PET-CT scans and bone marrow biopsy were the major methods applied to evaluate the lymphoma lesions. The response assessment criteria were as follows: (1) CR, absence of clinical symptoms, PET-CT, and bone marrow evidence associated with lymphoma; (2) PR, lymphoma volume decreases at least 50% without new lymphoma lesions or sustained bone marrow involvement; (3) PD, lymphoma volume increases at least 50% or onset of new lymphoma lesions; and (4) SD, a condition achieving neither the criteria for CR, PR, nor PD.

## FDG PET/CT Imaging and Calculation of PET/CT Metabolic Parameters

The process of the ^18^F-FDG PET/CT test was described previously (5). In brief, the ^18^F-FDG radioisotopes were produced by Siemens Eclipse cyclotron (Siemens Medical Solutions, Knoxville, TN, USA) and FDG4 chemical module. Then, the ^18^F-FDG solution was injected intravenously with a dosage of 4.44–5.55 MBq/kg. The craniocaudal scans were taken nearly 1–1.5 h after injection. It is worth mentioning that the patients were required to fast for at least 6 h before the scans. Then, the images were obtained through Siemens PET/CT Biograph 16 (Siemens Medical Solutions, Knoxville, TN, USA). The PET/CT images were reviewed visually by two experienced nuclear medicine physicians, and the inconsistency was determined by a consensus between these two readers. The metabolic parameters like MTV, TLG, and SUV were calculated using the Syngo-volume-counting program (Siemens Medical Solutions, Knoxville, TN, USA). In this study, all patients underwent baseline ^18^F-FDG PET/CT scans before and after CAR-T cell therapy to evaluate their disease conditions.

### Statistical Analysis

This study was designed to determine the association of the PET/CT prognostic parameters (MTV, TLG, and SUV_avg_) and CAR-T cell therapy’s safety and efficiency in R/R NHL patients. This study’s primary endpoints were OS and PFS rates in R/R NHL patients after CAR-T cell therapy, whereas the secondary endpoint was response rate and the occurrence of CAR-T-related adverse events. Generally, the OS was measured from CAR-T cell infusion until death from any cause or the last follow-up date. Meanwhile, the PFS was calculated as the time between CAR-T cell infusion and first relapse or disease progression. The numeric variables were presented as median (range) and compared using the Mann–Whitney *U* test. The categorical variables were presented as number (%), and the chi-squared test or Fisher’s exact test was used for comparisons. Next, Spearman’s correlation analysis was employed to analyze the correlations between PET/CT metabolic parameters and serum cytokine levels. To evaluate the risk factors associated with OS and PFS, a Cox regression model was used to obtain the hazards ratio (HR) and 95% confidence intervals (CI). Moreover, the optimal cutoff value of baseline PET/CT metabolic parameters was determined by the receptor-operated curve (ROC). The area under the ROC curve (AUC) indicates the predictive power, and an AUC greater than 0.5 confirms the predictive ability statistically. Then, the survival analysis was done by the Kaplan–Meier method, and the differences between groups were compared using the log-rank test. The differences between groups were considered statistically significant at the *p*-values of <0.05 (two-sided). All analyses were performed using IBM SPSS Statistics (version 23; IBM Corp., Armonk, NY).

## Results

### Patient Characteristics and PET/CT Metabolic Parameters

A total of 41 patients with R/R NHL treated with CD19 CAR-T cell therapy were included in this study. The patients’ characteristics of both the CR group and non-CR group are displayed in **Table 1**. According to data collected, the CR and non-CR groups’ median age was 44 years (range: 25–71) and 55 years (range: 22–70), respectively. As for the disease baseline indicators, the CR group showed a lower proportion of stage IV disease than the non-CR group (10/24, 41.7% *vs.* 13/17, 76.5%, *p* = 0.027). Besides, the CR group achieved a significantly higher PFS rate (11/22, 50% *vs.* 4/15, 26.7%, *p* = 0.042) and survival rate (18/22, 81.8% *vs.* 7/17, 41.2%, *p* = 0.009) than the non-CR group. Additionally, the adverse events and clinical outcomes were determined and compared between different groups of patients according to the PET/CT metabolic parameters. The patient groups were classified based on the optimal cutoff values for different prognostic parameters. According to the maximum Youden index in the ROC curve, the cutoff values of baseline SUV_avg_, MTV, and TLG associated with OS were 4.36, 26.37cm^3^, and 78.61. In contrast, the optimal cutoff values of early post-therapy SUV_avg_ (SUV_E_), MTV (MTV_E_), and TLG (TLG_E_) related to PFS were 2.60, 0.55 cm^3^, and 1.54, respectively.

**Table 1:** Patients’ characteristics.

	CR group (*N* = 24)	Non-CR group (*N* = 17)	*p*-value
**Characteristics**			
**Age, years**	44 (25, 71)	55 (22, 70)	0.168
**Gender, *n* (%)**			0.187
**Male**	12 (50.0%)	12 (70.6%)	
**Female**	12 (50.0%)	5 (29.4%)	
**No. of prior therapy**	8 (2, 18)	4 (2, 14)	**0.044**
**No. of prior relapse**	1 (0, 3)	1 (0, 2)	0.689
**No. of extra lymphadenopathy**	1.5 (0, 5)	2 (0, 7)	0.350
**LDH before lymphodepletion**	226 (133, 943)	253 (123, 576)	0.575
**Refractory type, *n* (%)**			0.698
**Primary refractory**	4 (16.7%)	4 (23.5%)	
**Refractory to second line or relapse after Auto-HSCT**	20 (83.3%)	13 (76.5%)	
**Risk stratification according to IPI score, *n* (%)**			0.103
**Low-risk**	9 (37.5%)	2 (11.8%)	
**Intermediate-risk**	14 (58.3%)	12 (70.6%)	
**High-risk**	1 (4.2%)	3 (17.6%)	
**Ann Abor staging, *n* (%)**			**0.027**
**Stage II–III**	14 (58.3%)	4 (23.5%)	
**Stage IV**	10 (41.7%)	13 (76.5%)	
**CAR-T cell dose (×10^6^/kg)**	5.04 (0.56, 10)	5.4 (1.60, 10.76)	0.534
**Coagulopathy, *n* (%)**	20 (83.3%)	15 (88.2%)	0.904
**Baseline MTV (cm^3^)**	54.18 (0.02, 1,168.3)	119.37 (15.69, 1,108.8)	0.610
**Baseline TLG**	462.86 (0.04, 13,472.5)	605.79 (78.92, 9,269.6)	0.526
**Baseline SUV**	4.87 (1.8, 11.53)	5.54 (3.06, 11.83)	0.130
**Early MTV after CAR-T cell therapy**	0 (0.0, 951.46)	0.45(0, 6,460.78)	**0.030**
**Early TLG after CAR-T cell therapy**	0 (0.0, 5,765.85)	1.30 (0, 33,562.45)	**0.025**
**Early SUV after CAR-T cell therapy**	0 (0.0, 6.06)	2.94 (2.64, 5.24)	**0.018**
**Overall survival, *n* (%)**			**0.009**
**≤180 days**	22/22 (100%)	9/17 (52.9%)	**0.001**
**>180 days**	12/16 (75%)	6/8 (75%)	0.915
**Progression free survival, *n* (%)**			**0.042**
**≤180 days**	17/22 (77.3%)	4/16 (25.0%)	**0.001**
**>180 days**	6/12 (50%)	3/3 (100%)	0.136

The data were described as n (%) or median (range). p-values were tested by the chi-square test or Mann–Whitney U test. (LDH, lactate dehydrogenase; CRS, cytokine release syndrome; MTV, metabolic tumor volume; TLG, total lesion glycolysis; SUV, standardized uptake value). Bold values indicate that the differences between the two groups were statistically significant.

### Correlations Between Baseline PET/CT Metabolic Parameters and Prognostic Factors

Based on the NCCN guidelines, the NHL prognosis’s prediction methods comprised age, IPI score, risk stratification, Ki-67, lactate dehydrogenase (LDH), and β2-microglobulin prior to lymphodepletion. Herein, we assessed the correlations between these clinical indicators and baseline PET/CT metabolic parameters in order to evaluate the predictive power of SUV_avg_, MTV, and TLG (**Figure 2J**). The results showed that all the baseline PET/CT metabolic parameters (SUV_avg_, MTV, and TLG) were significantly associated with LDH (*R*_SUV_ = 0.543, *p*_SUV_ = 0.001; *R*_MTV_ = 0.459, *p*_MTV_ = 0.006; *R*_TLG_ = 0.482, *P*_TLG_ = 0.004) and Ki-67 (*R*_SUV_ = 0.586, *p*_SUV_ = 0.003; *R*_MTV_ = 0.452, *p*_MTV_ = 0.027; *R*_TLG_ = 0.465, *P*_TLG_ = 0.022). Besides, positive correlations were also observed between baseline SUV_avg_, TLG, and IPI score (*R*_SUV_ = 0.469, *p*_SUV_ = 0.003; *R*_TLG_ = 0.339, *P*_TLG_ = 0.040), and risk stratification of the disease (*R*_SUV_ = 0.431, *p*_SUV_ = 0.008; *R*_TLG_ = 0.331, *P*_TLG_ = 0.045).

### Baseline and Early Post-Treatment PET/CT Metabolic Parameters Associated With Treatment Outcomes

After a median follow-up of 7 months (range: 1.1 to 44 months), the overall response rate (ORR) obtained 1 month after CAR-T cell infusion was 92.7%; 58.5% (24/41) of the patients achieved a complete remission (CR), and 34.1% (14/41) of the patients achieved partial remission (PR). However, 2.4% (1/41) of the patients were unresponsive to CAR-T cell therapy, and 4.9% (2/41) of the patients experienced disease progression at the time of response assessment. Next, the median OS and PFS were 209 (range: 34 to 1,330) days and 132 (range: 23 to 1,127) days.

**Figure 1:**
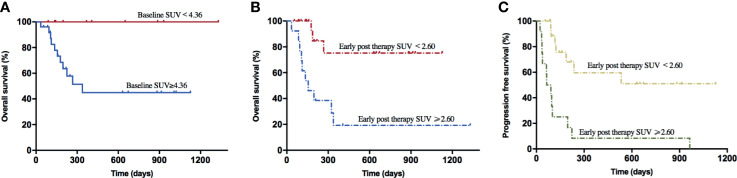
Survival curves of lymphoma patients associated with baseline SUV and early post-therapy SUV. **(A)** The 1-year OS rate of the baseline SUV < 4.36 group (100.0%) was higher than the baseline SUV ≥ 4.36 group (56.0%) (*p* = 0.019). **(B)** The 1-year OS rate of the early post-therapy SUV < 2.60 group (85.0%) was higher than the early post-therapy SUV ≥ 2.60 group (23.1%) (*p* = 0.001). **(C)** The PFS rate of the early post-therapy SUV < 2.60 group (65.0%) was higher than the early post-therapy SUV ≥ 2.60 group (0.0%) (*p* < 0.001).

The results of univariate analysis for OS and PFS using baseline patients’ characteristics and PET/CT metabolic parameters are presented in **Supplementary Table 1**. Furthermore, the multivariate analyses displayed that extra lymphadenopathy (HR: 1.708, 95% CI: 1.101–2.649; *p* = 0.017), the grading of CRS (HR: 2.322, 95% CI: 1.046–5.154; *p* = 0.038), and early post-therapy SUVavg (SUV_E_) (HR: 1.418, 95% CI: 1.029–1.955; *p* = 0.033) were the independent risk factors that associated with OS. In **Figure 1** and **Supplementary Figure 1**, the Kaplan–Meier analysis of survival demonstrated that the 1-year OS rate of patients with low baseline SUVavg were significantly higher than those in the high SUVavg group (100% *vs.* 44.9%, *p* = 0.019). Besides, the SUV_E_, MTV_E_, and TLG_E_ were negatively correlated to OS.

On the other hand, through the univariate analysis for PFS, we found that Ann Abor staging, SUV_E_, MTV_E_, and TLG_E_ after CAR-T cell therapy were significantly associated with inferior PFS. Besides, MTV_E_ (HR: 1.000, 95% CI: 1.001–1.002; *p* = 0.041) was identified as one of the risk factors for poor PFS through multivariate analyses. In addition, patients with SUV_E_ < 2.60 achieved a more extended 1-year PFS rate than those with SUV_E_ ≥ 2.60 (59.6% *vs.* 0.0%, *p* < 0.001); similar outcomes were observed in patient groups with different early post-therapy MTV (53.6% *vs.* 0.0%, *p* = 0.001) or TLG (53.6% *vs.* 0.0%, *p* = 0.001).

### Baseline PET/CT Metabolic Parameters Associated With Adverse Effects

**Figure 2:**
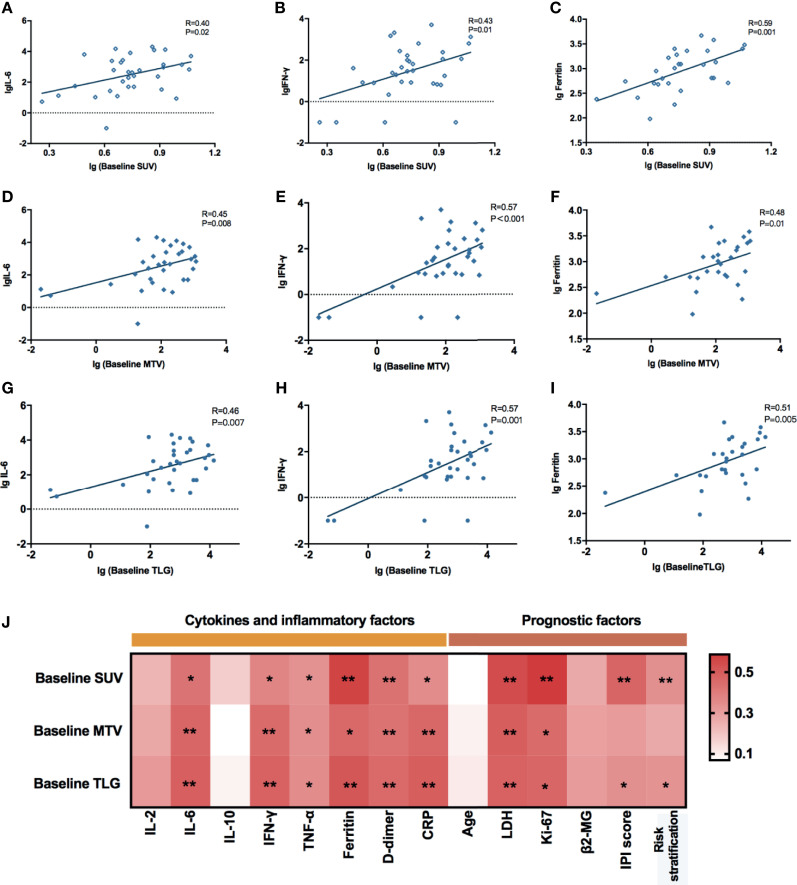
**(A–I)** Correlations between baseline PET/CT metabolic parameters and peak serum cytokines during CRS. All these values were given as lg. Linear regression was used for statistical analysis. **(A–C)** lg (Baseline SUV) were significantly correlated with lg (peak IL-6) (*R* = 0.40, *p* = 0.02), lg (peak IFN-γ) (*R* = 0.43, *p* = 0.01), and lg (peak Ferritin) (*R* = 0.59, *p* = 0.001). **(D–F)** lg (Baseline MTV) were significantly correlated with lg (peak IL-6) (*R* = 0.45, *p* = 0.008), lg (peak IFN-γ) (*R* = 0.57, *p* < 0.001), and lg (peak Ferritin) (*R* = 0.48, *p* = 0.01). **(G–I)** lg (Baseline TLG) were significantly correlated with lg (peak IL-6) (*R* = 0.46, *p* = 0.007), lg (peak IFN-γ) (*R* = 0.57, *p* = 0.001), and lg (peak Ferritin) (*R* = 0.51, *p* = 0.005). **(J)** Spearman correlation between baseline PET/CT metabolic parameters and prognostic factors of NHL. *0.01 ≤ P < 0.05, **0.001 ≤ P< 0.01.

**Figure 3:**
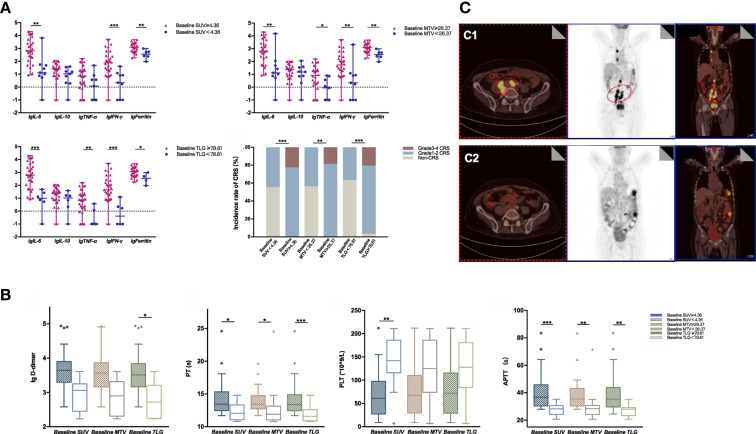
**(A)** lg (peak serum cytokine level) between different groups defined by the optimal cutoff values of baseline MTV, TLG, and SUV were compared using the Mann–Whitney *U* test. Data were described as median (range). High baseline SUV, MTV, and TLG group had significantly increased lg (peak serum cytokine level) than the compared groups; groups with baseline SUV ≥ 4.36 (*p* < 0.001), MTV ≥ 26.37 (*p* = 0.001), and TLG ≥ 78.61 (*p* < 0.001) had a higher incidence of severe CRS than the compared groups. **(B)** Indicators associated with coagulation function between different groups defined by the optimal cutoff value of baseline MTV, TLG, and SUV were compared using the Mann–Whitney *U* test. Data were described as median (95% CI). Groups with baseline TLG≥78.61 had significantly higher lg (peak D-dimer) than the compared group (P = 0.040); groups with baseline PET/CT metabolic parameters greater than or equal to the cutoff value had prolonged APTT and PT; the group with baseline SUV < 4.36 had higher PLT than the baseline SUV ≥ 4.36 group (*p* = 0.002). **(C)** Typical radiographic changes of PET/CT in a DLBCL patient after CAR-T cell therapy. **(C1)** Prior to CAR-T cell therapy, lymphoma cells involved in the abdominal aorta, right iliac vessels, as well as mediastinum exhibited high metabolic activity in FDG PET/CT; **(C2)** One month post CAR-T cell infusion, the metabolic activity of the abdominal aorta and right iliac vessels decreased significantly. *0.01 ≤ P < 0.05, **0.001 ≤ P < 0.01, ***P < 0.001.

The common adverse effects after CAR-T cell therapy are CRS, immune effector cell-associated neurotoxicity syndrome (ICANS), and coagulation disorders. CRS is a systemic inflammatory reaction syndrome caused by the release of cytokines. It usually manifests by clinical symptoms and signs like fever, tachypnea, headache, tachycardia, hypotension, rash, and hypoxia (16). In this research, 41 patients were enrolled and recruited. Of all, 82.9% (34/41) of the patients suffered from CRS; 14.6% (6/41) were reported with grade 3 CRS, yet no patient developed grade 4–5 CRS. Based on the univariate and multivariate analyses, the baseline SUV_avg_ (OR: 1.481, 95% CI: 1.030–2.130; *p* = 0.034) was an independent risk factor for CRS (**Table 2** and **Supplementary Table 1**). The correlations between PET/CT metabolic parameters and CRS’s clinical indicators are also shown in **Figure 2**. The Spearman correlation analyses revealed that baseline TLG was strongly related to peak value of IL-6 (*R* = 0.488, *p* = 0.003), IFN-γ (*R* = 0.478, *p* = 0.004), ferritin (*R* = 0.514, *p* = 0.004), and D-dimer (*R* = 0.455, *p* = 0.005). Moreover, the linear regression analysis between lg baseline PET/CT metabolic parameters and lg (peak IL-6), lg (peak IFN-γ), and lg (peak ferritin) exhibited a positive correlation between baseline tumor metabolic burden and serum cytokine concentration during CRS occurrence. Also, we assessed CRS occurrence and cytokine levels among different groups based on the baseline PET/CT metabolic parameters (**Figure 3A**). As a result, the groups with higher baseline MTV (5/26, 19% *vs.* 0/15, 0%; *p* = 0.001), TLG (6/29, 21% *vs.* 0/12, 0%; *p* < 0.001), and SUV_avg_ (6/26, 23% *vs.* 0/15, 0%; *p* < 0.001) tend to have an increased incidence of severe CRS than the other groups. Likewise, the former groups had higher peak serum cytokine levels than the groups with low baseline MTV, TLG, and SUV_avg_, and the differences in lg (peak IL-6), lg (peak IFN-γ), and lg (peak ferritin) between the groups were statistically significant. For instance, the difference in cytokine levels among the groups is portrayed in **Supplementary Table 2**.

**Table 2:** Multivariate analysis for risk factors associated with CRS, PFS, and OS.

Factors			Odds ratio (95% CI)	*p*-value	
**CRS **	Grade 0–2 *vs.* Grade 3–4			
Baseline SUV			1.481 (1.030, 2.130)	0.034	
			**Hazard ratio (95% CI)**		
**PFS after CAR-T cell therapy**					
MTV_E_ after CAR-T cell therapy			1.001 (1.000, 1.002)	0.041	
**OS after CAR-T cell therapy**					
No. of extra lymphadenopathy			1.708 (1.101, 2.649)	0.017	
Grade of CRS			2.322 (1.046, 5.154)	0.038	
SUV_E_ after CAR-T cell therapy			1.418 (1.029, 1.955)	0.033	

The data were described as hazard ratio (95% CI). The Cox regression model tests were used to test the p-values. (CRS, Cytokine release syndrome; PFS, Progression-free survival; OS, Overall survival; MTV_E_, early post therapy MTV; SUV_E_, early post therapy SUV).

Apart from CRS, coagulation disorders are another common side effects of CAR-T cell therapy. Coagulation disorders are associated with the endothelial system’s activation or injury, accompanied by prolonged prothrombin time (PT) and activated partial prothrombin time (APTT). Also, CAR-T patients with coagulation disorders might have an elevated D-dimer level and a low platelet count, which may eventually lead to vital visceral hemorrhage. Our study showed that 85.4% (35/41) of the patients experienced coagulation disorders. As displayed in **Supplementary Table 3** and **Figure 3B**, patients with higher baseline PET/CT metabolic parameters experienced a higher risk of coagulation disorder. For instance, they had an increased D-dimer level, prolonged APTT, PT, and INR than those with lower tumor burdens. To explain further, APTT and PT reflected the function of intrinsic and extrinsic coagulation pathways. In the groups with baseline MTV ≥ 26.37 cm^3^, baseline SUV_avg_ ≥ 4.36, and baseline TLG ≥ 78.61, the APTT and PT were dramatically lengthened.

### Typical Radiographic Changes of PET/CT After CAR-T Cell Therapy

PET/CT scans are indispensable in evaluating lymphoma lesions and therapeutic responses of lymphoma patients. In this paper, we also displayed the typical radiographic changes of PET/CT of a patient who had successfully obtained CR after receiving CAR-T treatment (**Figure 3C**). This 67-year-old male patient was diagnosed with diffuse large B-cell lymphoma and had a relapse after 13 cycles of chemotherapy. Ten days before the CAR-T therapy, his FDG PET/CT scans revealed multiple lymphadenopathies with high metabolic activity surrounding the abdominal aorta, right iliac vessels, and mediastinum. The most extensive lesion was at retroperitoneum, with a SUV_avg_ of 5.59, MTV of 394.18 cm^3^, and TLG of 2,203.47 (**Figure 3C1**). One month after CAR-T cell infusion, the PET/CT result showed a markedly reduced tumor volume with decreased FDG in the abdominal aorta and right iliac vessels. The hypermetabolic lesion in the mediastinum had nearly disappeared (**Figure 3C2**).

## Discussion

Previous studies have affirmed the role of ^18^F-FDG PET/CT scans in predicting Hodgkin’s lymphoma (HL) and NHL patients’ disease prognosis. Due to their significances in disease prognosis prediction, SUV-based, MTV-based, or TLG-based parameters were proposed as the early identification of a dismal disease prognosis. A recent study assessed the early response to anti-PD-1 agents in HL patients based on PET-derived biomarkers. In the study, they observed that ΔMTV and ΔTLG might potentially quantify patients’ response to PD-1 inhibitors (17). Nonetheless, the predictive value of baseline and post-therapy PET/CT metabolic parameters in CAR-T cell therapy remains unclear. Hence, our study is valuable as it presented the role of SUV, MTV, and TLG in assessing OS as well as PFS of R/R NHL patients after receiving CAR-T cell therapy, and the optimal cutoff values of each metabolic parameter may be informative in estimating the treatment’s safety and effectiveness.

The baseline tumor burden has been proved to be closely related to the adverse events and prognosis in acute lymphoblastic leukemia patients after CAR-T cell therapy. Meanwhile, for NHL patients, PET/CT is widely used to determine and measure disease burdens. Nevertheless, due to the tumor microenvironment, tumor burden in NHL patients measured by FDG PET/CT scan may not reflect the actual number of tumor cells. Our study proved that the baseline SUV_avg_, MTV, and TLG were correlated with pre-lymphodepletion LDH as well as Ki-67, and these biomarkers exhibited reliable predictive power for OS and PFS. Also, we found that patients in the high baseline SUV_avg_ group had a relatively lower OS rate. Those patients with high baseline MTV and TLG also exhibited similar trends, but were not statistically significant, which might be attributed to the short follow-up time. In addition, SUV reflects the tumor metabolic activity; it is more strongly associated with the malignancy of the lymphoma lesions than MTV and TLG.

Because CAR-T cell therapy is a relatively new cell-based therapy, there are only limited existing studies to investigate the relationship of tumor burden and disease prognosis. In a previous study conducted by our center, there was no significant difference in baseline MTV and TLG between R/R NHL patients with or without response after CD19 CAR-T cell therapy. These two PET/CT metabolic parameters were not associated with OS. However, the study outcomes might be restricted to limited studies due to the small sampling size^5^. Conversely, Erin et al. demonstrated that the patients with baseline MTV < 147.5 ml had significantly longer OS and PFS after summarizing the treatment responses of 96 patients treated with axicabtagene ciloleucel (axi-cel) (12). In addition to the similar findings, we further suggested that lower early post therapy PET/CT parameters might serve as protective factors for superior survival. Patients with SUV_E_ < 2.6, MTV_E_ < 0.55 cm^3^, and TLG_E_ < 1.54 had prolonged OS and PFS. These findings indicated that complete remission (CR), which was achieved 1 to 2 months after CAR-T cell therapy, may portend a durable PFS. Therefore, we concluded that PET/CT plays an indispensable role in early post-therapy monitoring.

CRS and coagulation disorders are recognized as post-CAR-T cell therapy adverse events. Many studies suggested that CRS were interlinked with CAR T cells, tumor cells, mononuclear/macrophage system, and endothelial cells. CRS commonly develops systemic inflammation symptoms in response to CAR T cells’ binding with specific antigens on tumor cells, which subsequently stimulate the monocytes and secrete many cytokines (13). In the extensive multicenter CAR-T studies for R/R NHL (ZUMA-1, JULIET, and TRANSCEND), CRS’s severity was associated with tumor burden. For example, a 2-year follow-up of ZUMA-1 suggested that axicabtagene ciloleucel can induce 11% (12/108) of ≥ grade 3 CRS and 32% (35/108) ≥ grade 3 neurological toxicity (NT) (18). Next, in the JULIET international study involving 95 R/R LBCL patients treated with tisagenlecleucel, the incidence of grade 3 or 4 CRS, NT, and cytopenia that lasted more than 28 days were 22%, 12%, and 32%, respectively (19). Besides, among 269 Lisocabtagene maraleucel (liso-cel) patients in the TRANSCEND trial, grade 3 or worse CRS and NT have occurred in 2% (6/269) and 10% (27/269) of the patients, respectively. Those patients with high tumor burden or increased inflammatory markers experienced a higher CRS incidence and NT (any grade) (20). Moreover, a high tumor burden (high MTV) was also a risk factor related to grade 3–4 CRS (5, 12). In line with these findings, our studies further indicated that high tumor burden reflected by baseline SUV_avg_ was a significant risk factor of grade 3–4 CRS. The patients with baseline SUV_avg_ ≥ 4.36, MTV ≥ 26.37 cm^3^, and TLG ≥ 78.6 were more likely to develop any grade of CRS and had increased levels of serum cytokines.

On the other hand, coagulation disorders are often complicated with severe CRS. Several reports proposed that endothelial activation triggered by many cytokines during severe CRS may be an underlying mechanism for coagulation disorders (21, 22). To validate, Wang and colleagues carried out a comprehensive analysis of coagulation parameters in 100 patients with R/R hematologic malignancies after CAR-T cell therapy. They observed a high incidence of coagulation disorders in these patients, including an elevated level of D-dimer (50/100, 50%), prolonged APTT (16/100, 16%), and prolonged PT (10/100, 10%). The occurrence and severity of coagulation disorders were positively associated with high tumor burden and the CRS progression in ALL patients (23). In short, our study confirmed the earlier finding as the coagulation parameters were remarkably higher in patients with higher baseline SUV_avg_, MTV, and TLG.

We are aware that our study may have several limitations. Firstly, it is a retrospective single-center study with a small sample size. Secondly, we also failed to evaluate the relationship between baseline PET/CT metabolic parameters and neurotoxicity due to the low incidence of ICANS in our CAR-T patients. Further data collection and multicenter clinical studies with a larger sample size would be needed to determine and develop a combined assessment system based on SUV, MTV, and TLG by ^18^F-FDG PET/CT scan. It may effectively and precisely predict the tumor burdens of R/R NHL patients before and after CAR-T cell therapy.

In conclusion, our study validated PET/CT scan’s role in predicting efficacy and adverse effects of CAR-T cell therapy in NHL. The study outcomes also demonstrated that heavy baseline tumor burdens might serve as the risk factors of CRS and coagulation disorders in R/R NHL patients after CAR-T cell infusion. Furthermore, lower early post-therapy SUV, MTV, and TLG values may enhance the OS and PFS of R/R NHL patients. Therefore, a comprehensive assessment system of tumor burdens based on PET/CT metabolic parameters may play a crucial role in guiding the tumor-debulking regimen before CAR-T cell infusion and managing adverse events during CAR-T cell therapy. Further studies on improving lymphodepletion chemotherapy, CRS prevention, and control are required to enhance CAR-T cell therapy’s therapeutic efficacy and safety in R/R NHL patients.

## Data Availability Statement

The original contributions presented in the study are included in the article/**Supplementary Material**. Further inquiries can be directed to the corresponding authors.

## Ethics Statement

The studies involving human participants were reviewed and approved by Research Ethics Committee of the First Affiliated Hospital, Zhejiang University School of Medicine. The patients/participants provided their written informed consent to participate in this study.

## Author Contributions

KZ, HH, and YH designed and oversaw the study. RH and ET were responsible for writing the manuscript. RH and LW collected and analyzed the data. XZ and GWa contributed to the PET-CT testing. All authors contributed to the article and approved the submitted version.

## Funding

This work was supported by grants from the Natural Science Foundation of China (grant nos. 81730008 and 81770201) and the Key Project of Science and Technology Department of Zhejiang Province (grant nos. 2019C03016 and 2018C03016-2).

## Conflict of Interest

The authors declare that the research was conducted in the absence of any commercial or financial relationships that could be construed as a potential conflict of interest.

## Publisher’s Note

All claims expressed in this article are solely those of the authors and do not necessarily represent those of their affiliated organizations, or those of the publisher, the editors and the reviewers. Any product that may be evaluated in this article, or claim that may be made by its manufacturer, is not guaranteed or endorsed by the publisher.
