# Supplementary material for: Tumor Burden Measured by 18F-FDG PET/CT in Predicting Efficacy and Adverse Effects of Chimeric Antigen Receptor T-Cell Therapy in Non-Hodgkin Lymphoma
Source: Front Oncol. 2021 Aug 4;11:713577. doi: 10.3389/fonc.2021.713577 (PMC8371710; doi:10.3389/fonc.2021.713577)
Supplement: Supplementary file 1 [file DataSheet_1.docx]

Supplementary Material

# Supplementary Figures and Tables

## Supplementary Figures


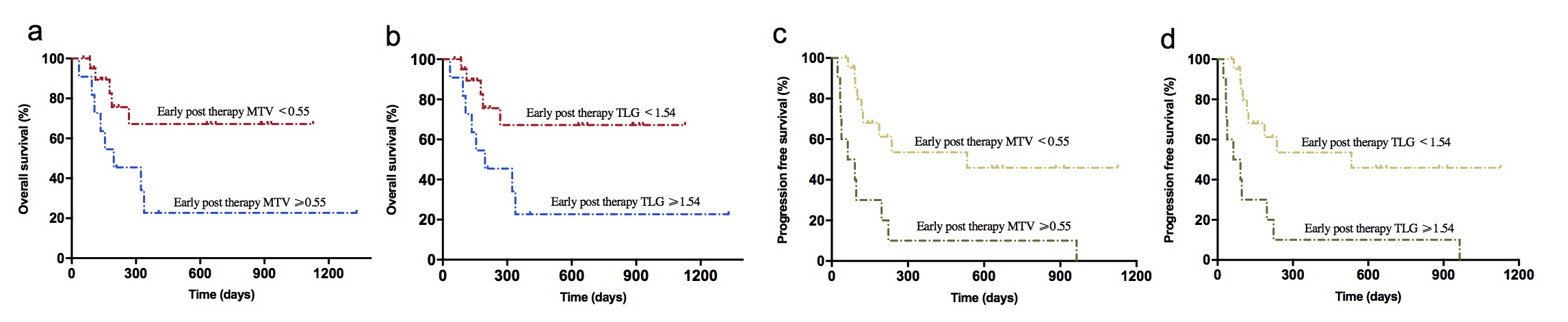


**Supplementary Figure 1.** Survival curves of lymphoma patients associated with early post-therapy PET-CT metabolic parameters. **a.** The 1-year OS rate of the early post-therapy MTV＜0.55 (64.8%) group was higher than the early post-therapy MTV≥0.55 group (22.7%) (P=0.042). **b.** The 1-year OS rate of the early post-therapy TLG＜1.54 (67.2%) group was higher than the early post-therapy TLG≥1.54 group (22.7%) (P=0.028). **c.** The 1-year PFS rate of the early post-therapy MTV＜0.55 group (53.6%) was higher than the early post-therapy MTV≥0.55 group (0.0%) (P=0.001). **d.** The 1-year PFS rate of the early post-therapy TLG＜1.54 group (53.6%) was higher than the early post-therapy TLG≥1.54 group (0.0%) (P=0.001).

**Supplement Table 1. Univariate analyses for factors impacting CRS, PFS and OS**

| **Factors** |  | **Hazard ratio (95% CI)** | **P-value** |
| --- | --- | --- | --- |
| ***CRS*** |  |  |  |
| Gender |  | 1.500 (0.264, 8.523) | 0.647 |
| Age |  | 0.978 (0.916, 1.044) | 0.500 |
| No. of prior therapy |  | 0.718 (0.497, 1.038) | **0.079** |
| No. of prior relapse |  | 0.121 (0.014, 1.028) | **0.053** |
| No. of extralymphadenopathy |  | 1.524 (0.910, 2.555) | 0.110 |
| LDH before lymphodepletion (U/L) |  | 1.001 (0.995, 1.006) | 0.842 |
| β2-MG before lymphodepletion (ug/L) |  | 0.999 (0.997, 1.000) | **0.069** |
| Ann Abor staging | Stage II vs. Stage III. Stage IV | 0.960 (0.223, 4.134) | 0.956 |
| IPI scores |  | 3.607 (0.958, 13.589) | **0.058** |
| CAR T-cell dose (×106/kg) |  | 1.342 (0.932, 1.932) | 0.114 |
| Baseline MTV (cm3) |  | 1.001 (0.999, 1.003) | 0.356 |
| Baseline TLG |  | 1.000 (1.000, 1.000) | 0.237 |
| Baseline SUV |  | 1.481 (1.030, 2.030) | **0.034** |
| ***Progression free survival*** |  |  |  |
| Gender |  | 0.950 (0.407, 2.215) | 0.905 |
| Age |  | 0.977 (0.944, 1.012) | 0.194 |
| No. of prior therapy |  | 0.932 (0.830, 1.047) | 0.234 |
| Refractory type | Primary refractory vs. Refractory  to second line or relapse after Auto-HSCT | 0.735 (0.286, 1.888) | 0.522 |
| Auto-HSCT before CAR-T cell therapy |  | 1.576 (0.613, 4.054) | 0.345 |
| No. of prior relapse |  | 0.634 (0.318, 1.264) | 0.196 |
| No. of extralymphadenopathy |  | 1.602 (1.212, 2.118) | **0.001** |
| LDH before lymphodepletion (U/L) |  | 1.002 (0.999, 1.004) | 0.139 |
| β2-MG before lymphodepletion (ug/L) |  | 1.000 (0.999, 1.000) | 0.769 |
| Ann Abor staging | Stage II vs. Stage III. Stage IV | 3.251 (1.235, 8.554) | **0.017** |
| IPI scores |  | 1.429 (0.943, 2.167) | **0.088** |
| CAR T-cell dose (×106/kg) |  | 1.019 (0.858, 1.210) | 0.830 |
| Treatment response | CR vs. PR+NR | 0.426 (0.183, 0.993) | **0.048** |
| Grade of CRS |  | 1.140 (0.697, 1.865) | 0.600 |
| Baseline MTV (cm3) |  | 1.000 (0.999, 1.002) | 0.736 |
| Baseline TLG |  | 1.000 (1.000, 1.000) | 0.873 |
| Baseline SUV |  | 0.947 (0.784, 1.143) | 0.569 |
| Early MTV after CAR-T cell therapy |  | 1.001 (1.000, 1.002) | **0.041** |
| Early TLG after CAR-T cell therapy |  | 1.000 (1.000, 1.000) | **0.021** |
| Early SUV after CAR-T cell therapy |  | 1.324 (1.061, 1.652) | **0.013** |
| ***Overall survival*** |  |  |  |
| Gender |  | 0.395 (0.122-1.280) | 0.121 |
| Age |  | 0.979 (0.940, 1.020) | 0.308 |
| No. of prior therapy |  | 0.854 (0.724-1.009) | **0.064** |
| Refractory type | Primary refractory vs. Refractory  to second line or relapse after Auto-HSCT | 0.507 (0.169, 1.518) | 0.225 |
| Auto-HSCT before CAR-T cell therapy |  | 1.098 (0.305, 3.952) | 0.887 |
| No. of prior relapse |  | 0.396 (0.155, 1.009) | **0.052** |
| No. of extralymphadenopathy |  | 1.934 (1.298, 2.908) | **0.001** |
| LDH before lymphodepletion (U/L) |  | 1.002 (0.999, 1.004) | 0.250 |
| β2-MG before lymphodepletion (ug/L) |  | 1.000 (0.999, 1.001) | 0.922 |
| Ann Abor staging | Stage II vs. Stage III. Stage IV | 3.231 (0.901, 11.584) | **0.072** |
| Risk stratification | Low- vs. Intermediate- vs. High-risk | 2.912 (1.157, 7.330) | **0.023** |
| CAR T-cell dose (×106/kg) |  | 1.154 (0.923, 1.443) | 0.208 |
| Treatment response | CR vs. PR+NR | 0.239 (0.074, 0.767) | **0.016** |
| Grade of CRS |  | 2.496 (1.209, 5.152) | **0.013** |
| Baseline MTV (cm3) |  | 1.000 (0.998, 1.002) | 0.900 |
| Baseline TLG |  | 1.000 (1.000, 1.000) | 0.876 |
| Baseline SUV |  | 1.069 (0.863, 1.324) | 0.541 |
| Early MTV after CAR-T cell therapy |  | 1.000 (1.000, 1.001) | **0.002** |
| Early TLG after CAR-T cell therapy |  | 1.000 (1.000, 1.000) | **0.002** |
| Early SUV after CAR-T cell therapy |  | 1.498 (1.128, 1.991) | **0.005** |

**Supplementary Table 1.** Data were expressed as Hazard ratio (95%CI). P values were tested by the Cox regression model test. (Abbreviations: LDH: lactate dehydrogenase; CRS: cytokine release syndrome; Auto-HSCT: autologous hematopoietic stem cell transplantation; MTV: metabolic tumor volume; TLG: total lesion glycolysis; SUV: standardized uptake value; β2-MG: β2-microglobulin.)

**Supplement Table 2. Mann-Whitney U test for PEC/CT metabolic parameters and clinical indicators of CRS**

| **clinical indicators** | **Baseline MTV≥ 26.37 cm^3^ group**  **(N=26)** | **Baseline MTV＜26.37 cm^3^ group**  **(N=11)** | **P value** |  |
| --- | --- | --- | --- | --- |
| Maximum IL-2, pg/ml | 3.4 (1.4, 7.1) | 1.4 (0.1, 4.6) | **0.080** |  |
| Maximum IL-6, pg/ml | 645.6 (161.2, 3335.9) | 13.1 (6.4, 80.9) | **0.002** |  |
| Maximum IL-10, pg/ml | 23.8 (7.7, 41.8) | 15.3 (4.6, 36.8) | 0.492 |  |
| Maximum IFN-γ, pg/ml | 54.5 (15.2, 249.6) | 2.2 (0.1, 10.6) | **0.005** |  |
| Maximum TNF- α, pg/ml | 8.2 (1.7, 30.2) | 0.9 (0.1, 5.5) | **0.016** |  |
| Maximum ferritin, ng/ml | 1228.2 (627.6, 2313.2) | 380.8 (205.2, 625.6) | **0.003** |  |
| Maximum CRP, mg/L | 82.7 (31.1, 150.2) | 6.5 (3.1, 24.5) | **＜0.001** |  |
| Maximum D-dimer, ng/ml | 3692.0 (1468.8, 6628.0) | 1205.5 (290.5, 2702.8) | **0.015** |  |
|  | **Baseline TLG ≥ 78.61 group**  **(N=29)** | **Baseline TLG＜78.61 group**  **(N=8)** |  |  |
| Maximum IL-2, pg/ml | 3.6 (1.4, 7.3) | 0.3 (0.1, 1.7) | **0.003** |  |
| Maximum IL-6, pg/ml | 613.0 (84.5, 3897.6) | 10.3 (4.0, 32.7) | **＜0.001** |  |
| Maximum IL-10, pg/ml | 25.3 (7.8, 41.8) | 11.2 (1.6, 22.1) | 0.122 |  |
| Maximum IFN-γ, pg/ml | 49.0 (7.8, 257.2) | 0.9 (0.1, 4.7) | **＜0.001** |  |
| Maximum TNF- α, pg/ml | 7.3 (1.6, 22.8) | 0.1 (0.1, 1.8) | **0.005** |  |
| Maximum ferritin, ng/ml | 1194.0 (531.2, 2299.2) | 373.5 (131.8, 866.7) | **0.036** |  |
| Maximum CRP, mg/L | 80.5 (28.9, 148.2) | 7.1 (3.0, 21.7) | **0.001** |  |
| Maximum D-dimer, ng/ml | 3210.0 (1463.5, 7009.0) | 529.0 (175.0, 1611.0) | **0.002** |  |
|  | **Baseline SUV ≥ 4.36 group**  **(N=26)** | **Baseline SUV＜4.36 group**  **(N=11)** |  |  |
| Maximum IL-2, pg/ml | 4.2 (1.6, 7.3) | 0.5 (0.1, 1.7) | **0.002** |  |
| Maximum IL-6, pg/ml | 645.6 (175.1, 3335.9) | 13.12 (6.4, 53.1) | **0.001** |  |
| Maximum IL-10, pg/ml | 26.1 (7.8, 46.1) | 10.8 (2.0, 25.4) | 0.079 |  |
| Maximum IFN-γ, pg/ml | 76.0 (15.3, 360.6) | 2.2 (0.1, 10.3) | **＜0.001** | |
| Maximum TNF- α, pg/ml | 7.2 (1.3, 22.0) | 1.2 (0.1, 5.9) | 0.086 |  |
| Maximum ferritin, ng/ml | 99.1 (33.1, 150.2) | 381.9 (205.2, 657.3) | **0.006** |  |
| Maximum CRP, mg/L | 4441.5 (2018.5, 7945.5) | 10.3 (3.1, 24.5) | **＜0.001** |  |
| Maximum D-dimer, ng/ml | 2375.0 (1334.0, 5915.0) | 1152.5 (290.5, 1791.8) | **＜0.001** |  |

**Supplement Table 2.** Data were described as median (IQR). P-values were tested by the Mann-Whitney U test. (Abbreviations: CRP: C reactive protein; MTV: metabolic tumor volume; TLG: total lesion glycolysis; SUV: standardized uptake value.)

**Supplement Table 3. Mann-Whitney U test for PEC/CT metabolic parameters and clinical indicators of coagulation disorder**

|  | **Baseline MTV≥ 26.37 cm^3^ group**  **(N=26)** | **Baseline MTV＜26.37 cm^3^ group**  **(N=11)** | **P value** |
| --- | --- | --- | --- |
| Coagulation disorder, n(%) | 25 (96.2%) | 6 (54.5%) | **0.015** |
| Maximum PT, s | 13.5 (11.7, 19.7) | 11.9 (10.8, 24.6) | **0.010** |
| Maximum APTT, s | 35.4 (27.9, 83.5) | 28.4 (20.7, 71.7) | **0.005** |
| Maximum INR | 1.1 (1.0, 1.6) | 1.0 (0.9, 2.2) | **0.004** |
| Maximum D-dimer, ng/ml | 3692.0 (382.0, 88000.0) | 1205.5 (170.0, 80596.0) | **0.015** |
| Minimum PLT, *10^9/L | 67.5 (9.0, 212.0) | 125.0 (7.0, 211.0) | 0.094 |
|  | **Baseline TLG ≥ 78.6 group**  **(N=29)** | **Baseline TLG＜78.6 group**  **(N=8)** |  |
| Coagulation disorder, n(%) | 28 (96.6%) | 3 (37.5%) | **0.003** |
| Maximum PT, s | 13.4 (11.7, 24.6) | 11.6 (10.8, 14.8) | **0.007** |
| Maximum APTT, s | 35.3 (24.5, 83.5) | 28.0 (20.7, 35.2) | **0.002** |
| Maximum INR | 1.1 (1.0, 2.2) | 1.0 (0.9, 1.3) | **0.014** |
| Maximum D-dimer, ng/ml | 3210.5 (382.0, 88000.0) | 529.0 (170.0, 4055.0) | **0.002** |
| Minimum PLT, *10^9/L | 72.0 (9.0, 212.0) | 128.0 (7.0, 211.0) | 0.073 |
|  | **Baseline SUV ≥ 4.36 group**  **(N=26)** | **Baseline SUV＜4.36 group**  **(N=11)** |  |
| Coagulation disorder, n(%) | 25 (96.2%) | 6 (54.5%) | **0.015** |
| Maximum PT, s | 13.5 (11.7, 24.6) | 12.1 (10.8, 14.8) | **0.011** |
| Maximum APTT, s | 36.8 (27.9, 83.5) | 28.2 (20.7, 35.3) | **＜0.001** |
| Maximum INR | 1.1 (1.0, 2.2) | 1.1 (0.9, 1.3) | **0.012** |
| Maximum D-dimer, ng/ml | 4441.5 (382.0, 88000.0) | 1152.5 (170.0, 4055.0) | **＜0.001** |
| Minimum PLT, *10^9/L | 61.0 (9.0, 212.0) | 142.0 (7.0, 211.0) | **0.002** |

**Supplement Table 3.** Data were described as n (%) or median (range). P-values were tested by the Chi-Square test or Mann-Whitney U test. (Abbreviations: PT: prothrombin time; APTT: activated partial prothrombin time; INR: international normalized ratio; TLG: total lesion glycolysis; SUV: standardized uptake value.
